# A case series of forearm compartment syndrome complicating transradial cardiac catheterization

**DOI:** 10.1186/s43044-024-00498-y

**Published:** 2024-05-31

**Authors:** Nur Ayuni Khirul Ashar, Imma Isniza Ismail, Rahul Lingam, Naadira Faa’iza Mazlan, Nur Syahirah Azmi

**Affiliations:** 1 Orthopaedic Surgery & Traumatology Department, Faculty of Medicine, Universiti Teknologi MARA, Sungai Buloh, Selangor Malaysia; 2 Orthopaedic Department, Faculty of Medicine and Health Sciences, Universiti Putra Malaysia, Serdang, Selangor Malaysia; 3 Orthopaedic Department, Hospital Serdang, Serdang, Selangor Malaysia

**Keywords:** Compartment syndrome, Transradial catheterization, Fasciotomy, Case report

## Abstract

**Background:**

Acute compartment syndrome following a transradial coronary approach is rare. However, as the incidence of coronary arterial disease increases due to lifestyle factors and multiple comorbidities, transradial coronary angiography has become more common for diagnostic and therapeutic purposes in cardiovascular centers. Despite its rarity, we encountered two cases of acute compartment syndrome within a 1-week interval in the cardiology unit of a tertiary hospital.

**Case presentation:**

The first case involved a 75-year-old woman diagnosed with non-ST elevation myocardial infarction (NSTEMI). A coronary angiogram was performed via an uncomplicated right radial artery puncture. Following the procedure, the patient experienced significant swelling in the right forearm. An emergency fasciotomy release of the right forearm was conducted, revealing a gushing hematoma upon entering the flexor compartment. Fortunately, the wound healed well two months postoperatively with no functional deficits. In the second case, an 80-year-old man presented with severe angina pectoris upon exertion and was diagnosed with NSTEMI. The following day, he developed compartment syndrome in the left forearm, necessitating an emergency fasciotomy. Intraoperative examination revealed muscle bulging within the forearm compartments accompanied by extensive hematoma. Postoperatively, a deranged coagulation profile caused oozing from the wound. However, since there was no arterial bleeding, a compression dressing was applied. This led to a gradual drop in hemoglobin levels and worsened his heart condition. Despite resuscitative efforts and attempts to correct the coagulopathy, the patient experienced cardiorespiratory arrest and succumbed to ischemic heart disease in failure.

**Conclusion:**

Clinicians must remain vigilant in identifying this potentially limb-threatening condition. Patients with pre-existing anticoagulant therapy and underlying atherosclerotic disease are at a higher risk of bleeding complications. Implementing effective hemostasis techniques and promptly managing swelling can help prevent the occurrence of compartment syndrome. Timely assessment and maintaining a high level of clinical suspicion are paramount. If necessary, early consideration of decompressive fasciotomy is essential to avert catastrophic outcomes.

## Background

Acute compartment syndrome following a transradial coronary approach is rare, with an incidence of less than 0.01% [1]. It constitutes an orthopedic emergency, and failure to recognize it early may result in adverse outcomes. The most common causes include hematoma of fractured bones or fractures causing arterial or venous injury. Rarely, it can occur due to extravasation of intravenous fluids, intravenous antibiotics, or dye injections.

The transradial approach to cardiac catheterization is well-established among cardiologists and offers proven advantages over the femoral approach [2]. With the increasing incidence of coronary arterial disease due to lifestyle factors and multiple comorbidities, transradial coronary angiography has become more prevalent for diagnostic and therapeutic procedures in cardiovascular centers. Despite its rarity, we described two cases of acute compartment syndrome that occurred within a one-week interval in the cardiology unit of a tertiary hospital.

## Case presentation

### Case 1

A 75-year-old woman with a strong family history of ischemic heart disease presented to the casualty department with typical left-sided chest pain. She had underlying diabetes mellitus, hypertension, dyslipidemia, and chronic kidney disease. Diagnosis revealed a non-ST elevation myocardial infarction (NSTEMI), with a troponin I level of 1374 ng/L. The patient received loading doses of aspirin, clopidogrel, and subcutaneous low molecular weight heparin.

Preprocedural blood investigations indicated normal levels of hemoglobin, platelets, and coagulation screening. A coronary angiogram was performed via an uncomplicated right radial artery puncture using a 6-French sheath. Intraoperative anticoagulation was achieved with an intra-arterial bolus of 2000 IU of heparin at the beginning of the procedure. The angiogram revealed double-vessel disease with critical calcific stenosis of the right coronary artery. The cardiologist proceeded with urgent percutaneous coronary intervention, including stenting of the right coronary artery.

**Fig. 1:**
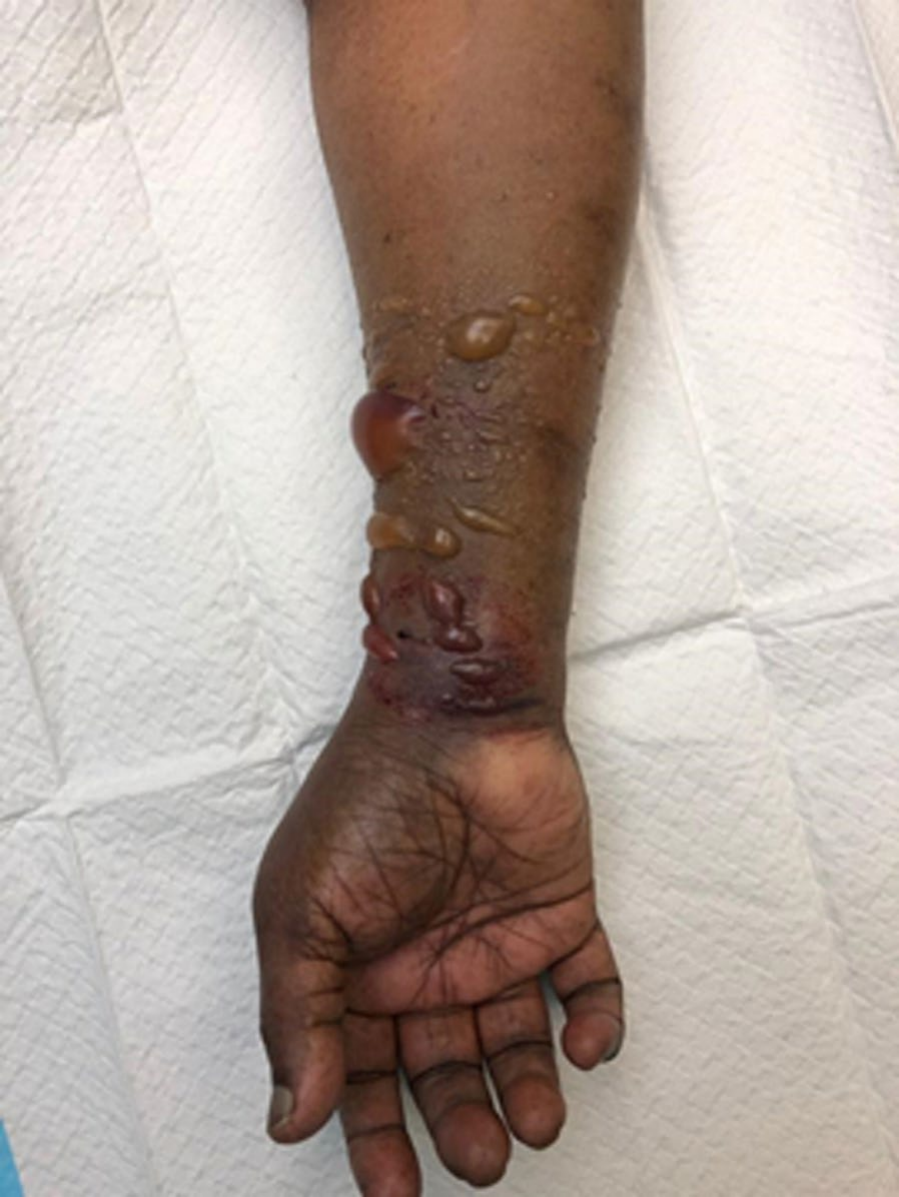
Pre-operative clinical picture of the right upper limb showing signs of acute compartment syndrome

Four hours postprocedure, a referral was made to the orthopaedic team upon noting gross swelling of the right forearm. Further history provided by the patient revealed that swelling over the right forearm had begun slowly and progressively after the procedure. The patient complained of increasing pain, which disturbed her sleep, and reported numbness in the fingers. Upon examination, gross swelling was observed over the volar aspect of the right forearm, with striking tense swelling over the distal half. Multiple bullous eruptions were noted over the volar aspect of the right forearm (Fig. 1). There was no active bleeding from the puncture site of the right radial artery at the wrist. The ulnar artery was palpable with strong volume; however, the radial artery could not be appreciated due to the gross swelling. A bedside Doppler ultrasound scan revealed a biphasic signal of the ulnar artery and a monophasic signal of the radial artery. Sensation was reduced over the fingertips, although oxygen saturation in all fingers ranged from 96 to 98%, with a negative passive stretch test. The signs and symptoms experienced by the patient were suggestive of compartment syndrome of the right forearm.

**Fig. 2:**
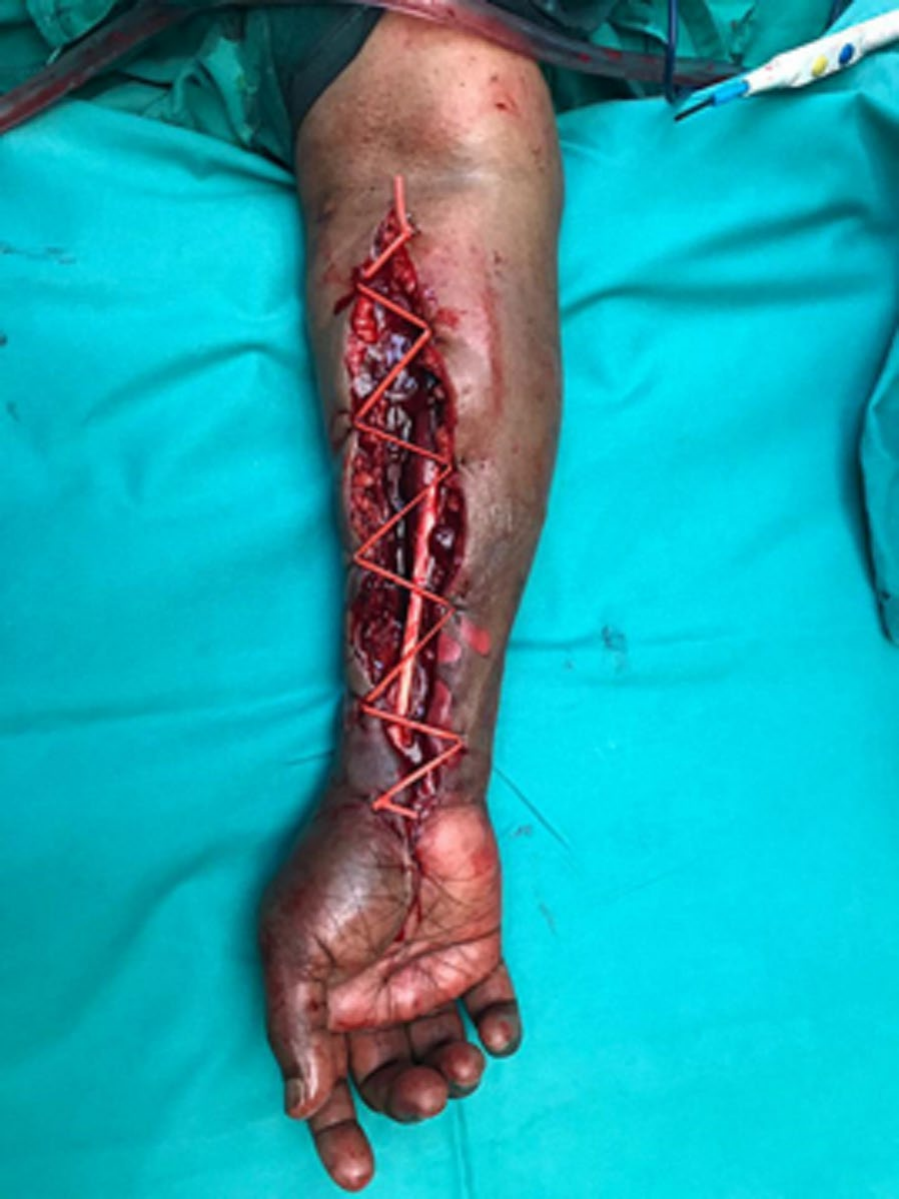
Postoperative clinical picture showing the shoelace technique used over the right forearm fasciotomy wound

After explaining the patient's condition to her and her family, she was taken to the operating theatre for emergency fasciotomy release of the right forearm, along with prophylactic right carpal tunnel release. Intraoperatively, upon opening the fascia over the volar forearm, muscle bulging from the volar and mobile wad compartments was observed. Gushing of hematoma was seen upon releasing the superficial and deep compartments of the right forearm. The muscles over these compartments were contused but viable. Both radial and ulnar pulses were strong and comparable after evacuating the hematoma. The surgeon opted to approximate the wound using the shoelace technique with a vessel loop (Fig. 2). Two months postoperatively, the wound healed well with no functional deficit.

### Case 2

An 80-year-old man with comorbidities of diabetes mellitus, hypertension, and dyslipidemia presented with severe angina pectoris upon exertion. His electrocardiogram showed sinus rhythm with no ST changes; however, troponin I levels were elevated at 142,200 ng/L, indicative of NSTEMI. He was initiated on subcutaneous heparin, oral aspirin, and clopidogrel for treatment.

The baseline blood tests revealed a prolonged activated partial thromboplastin time (APTT) of 76.2 s, with normal prothrombin time (PT) and International Normalized Ratio (INR) values of 16.3 s and 1.29, respectively. The full blood count was normal, with a hemoglobin level of 11.8 g/dL. Transradial coronary angiography was performed one day after admission, revealing three-vessel coronary artery disease. An intra-arterial bolus of 3000 IU of heparin was administered at the beginning of the procedure. The radial artery sheath was removed immediately after the completion of the intervention, and hemostasis was achieved by applying a compression dressing.

On the first day postprocedure while under cardio care, a referral was made to the orthopaedic team due to suspicion of impending compartment syndrome of the right forearm. Early assessment revealed mild swelling over the right hand and ipsilateral forearm, with all compartments otherwise feeling soft. Since there were no other signs or symptoms suggestive of compartment syndrome, the patient was advised to elevate the right forearm using an arm sling connected to a drip stand, and the swelling was carefully monitored.

**Fig. 3:**
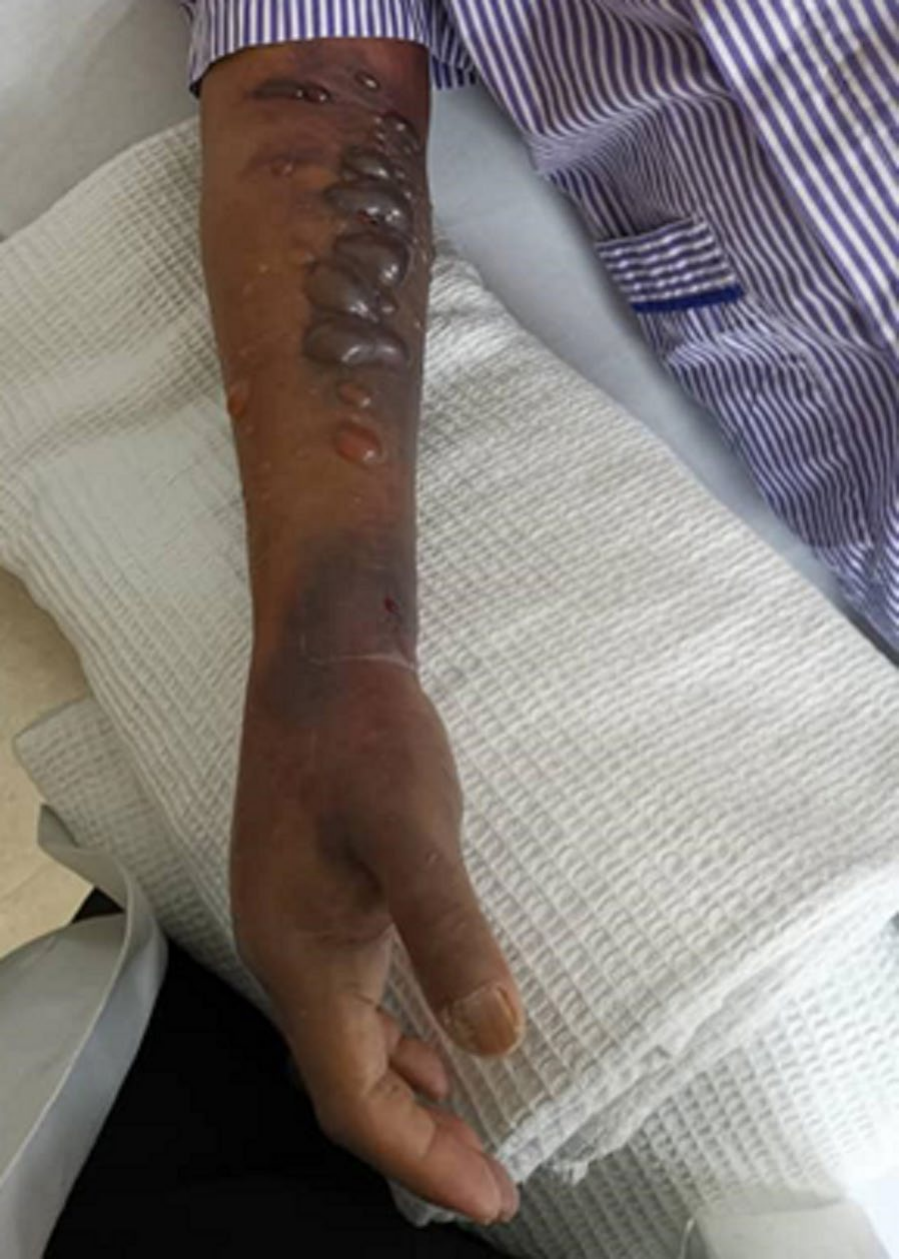
Grossly swollen right forearm with extensive bruising and blistering

Unfortunately, on the following day after our assessment, we observed worsening swelling of the right hand and forearm, accompanied by the formation of blisters and bruises over the forearm (Fig. 3). The compartments of the forearm were tense, while those of the hand remained soft. A passive stretch test was positive. Both radial and ulnar pulses were palpable with good volume. The range of motion of the small joints of the fingers, wrist, and elbow was restricted due to the swelling. At this point, a diagnosis of acute compartment syndrome was made, and the patient was promptly taken to the operating theatre for urgent volar and extensor compartment forearm fasciotomy, along with prophylactic carpal tunnel release under general anesthesia. Intraoperative findings revealed muscle bulging upon entering the forearm compartments, accompanied by abundant hematoma. However, the muscles were otherwise viable with good contractility.

After the procedure, a deranged coagulation profile led to oozing from the wound. However, as there was no active arterial bleeding, a compression dressing was applied. Unfortunately, the patient's hemoglobin level dropped slowly to 6.6 g/dL, exacerbating his heart condition. Despite resuscitative efforts and attempts to correct the coagulopathy, the patient experienced cardiorespiratory arrest one day after the procedure and passed away due to heart failure from ischemic heart disease.

## Discussion

Acute compartment syndrome manifests due to elevated intracompartmental pressure within an enclosed, non-expandable fascial compartment. It can occur with or without known trauma. Diagnosis of acute compartment syndrome relies on a combination of signs and symptoms, primarily assessed through clinical examination and the judgment of healthcare providers. This often leads to timely surgical intervention, guiding patients to the operating room. In some cases, direct measurement of tissue pressures within the compartment can aid in diagnosis and should be considered for patients at high risk for acute compartment syndrome as it has high sensitivity and specificity [3]. Patients typically present with gross and tense swelling and exhibit the '5 P's': acute, disproportionately worsening pain (pain not alleviated by analgesia), decreased motor function (paralysis), decreased sensation (paraesthesia), paleness, and pulselessness. The latter two are late sequelae, indicating that irreversible damage may have already occurred by that stage. Additionally, blistering and extensive bruising may also be observed.

Diagnosis is typically made when the intracompartmental pressure exceeds 30 mmHg, whereas normal pressure is typically less than 8 mmHg [4]. Alternatively, diagnosis can be based on a difference of less than 30 mmHg between systolic blood pressure and compartment pressure. The increase in pressure compresses the arteries supplying the tissue, leading to reduced tissue perfusion, tissue ischemia, and ultimately necrosis. Some tissues, including muscle and nerve, are more sensitive to ischemia. Both can tolerate ischemic events for up to 4 h, but irreversible damage occurs after 8 h [5].

Most cardiologists prefer the transradial approach for coronary catheterization over the transfemoral approach for several reasons. While the vascular complications of both approaches, including hematoma, arteriovenous fistula, vasospasm, pseudoaneurysm, and perforation [6] are similar, a higher percentage of complications is observed with transfemoral catheterization. This increased morbidity is attributed to the involvement of a larger caliber vessel, which serves as the primary blood supply to the lower limb. Due to the lower incidence and severity of complications, transradial catheterization typically results in a shorter hospital stay. Nowadays, there is an increased prevalence of young patients aged 40 or younger with cardiovascular diseases who need to undergo coronary angiography [7, 8]. Young adults, especially men, tend to develop compartment syndrome due to the larger muscle mass contained within fascial compartments [9]. Therefore, it is important to watch out for compartment syndrome in this age group postangiography. It is also important to note that the risk for compartment syndrome is higher in the forearm compared to the proximal thigh, although acute compartment syndrome of the forearm is less common than in the leg [9].

Acute compartment syndrome following a transradial approach for coronary angiography typically occurs due to bleeding or hematoma [10, 11]. Rarely, it can also result from spasm of the vessel itself [12]. Due to the location of the radial artery in the flexor compartment of the forearm, this compartment is usually the first to be affected.

The potential cause of bleeding in these cases is likely due to pre-existing anticoagulant therapy and the additional anticoagulation given for the acute episode of myocardial infarction. Supratherapeutic doses of anticoagulants administered pre-procedure may also play a role [1]. It is well known that anticoagulants used for the acute treatment of myocardial infarction and pre-procedural care help prevent further thrombosis. However, the accumulation of doses during the procedure may contribute to continuous oozing of blood into the compartments, even after compression. Moreover, atherosclerotic vessels may fail to contract after catheterization, leading to continuous leakage of blood into the compartments, especially after multiple failed cannulations [13].

To prevent compartment syndrome in such cases, ultrasonography can be beneficial for assessing the radial artery, reducing the need for multiple attempts at arterial puncture [14]. It is crucial to secure hemostasis with sufficient manual finger compression at the access site until bleeding has ceased [15]. Longer compression times may be necessary for patients on uninterrupted oral or intravenous anticoagulation. Frequent monitoring of the access site in the first 30 min after the procedure is essential. Early swelling management with forearm elevation and ice compression can also be effective. Additionally, mechanical compression is another option. While both manual and mechanical methods yield similar outcomes, manual compression offers the advantage of shorter hemostasis time. However, it requires a larger postprocedural care team for management [16].

Early prevention measures can help avoid the need for urgent fasciotomy, particularly given the higher risk for patients to undergo general anesthesia following a recent cardiac event. However, if the patient's condition deteriorates, urgent fasciotomy should not be delayed. A delay in diagnosis can lead to permanent dysfunction, and releasing the compartment at a late stage can result in a poorer outcome [17] and potential medicolegal cases [18].

## Conclusion

Although the incidence of acute compartment syndrome of the forearm following a transradial approach for coronary procedures is rare, clinicians must remain vigilant in recognizing this potentially limb-threatening condition. Patients with pre-existing anticoagulant therapy and underlying atherosclerotic disease are at a higher risk of bleeding complications. Effective hemostasis and swelling management are essential for preventing compartment syndrome. Timely assessment and maintaining a high level of clinical suspicion are paramount. If indicated, early decompressive fasciotomy should be considered to prevent any catastrophic outcomes.

## Abbreviations

NSTEMI: Non-ST Elevation Myocardial Infarction
APTT: Activated Partial Thromboplastin Time
PT: Prothrombin time
INR: International Normalized Ratio
